# Development and Validation of the Random Forest Model via Combining CT-PET Image Features and Demographic Data for Distant Metastases among Lung Cancer Patients

**DOI:** 10.1155/2022/7793533

**Published:** 2022-12-13

**Authors:** Lijun Bi, Yi Guo

**Affiliations:** ^1^Department of Electronic Information, Shandong University of Science and Technology (SDUST), Qingdao 266590, Shandong, China; ^2^Department of Radiology, Changzhou No. 2 People's Hospital, Changzhou 213000, Jiangsu, China

## Abstract

The work aimed at developing and validating a random forest model of CT-PET image features combined with demographic data to diagnose distant metastases among lung cancer patients. This study involved lung cancer patients from The Cancer Genome Atlas lung adenocarcinoma (TCGA-LUAD) dataset, the lung PET-CT dataset, the lung squamous cell carcinoma (LSCC) dataset, and the National Cancer Institute's Clinical Proteomic Tumor Analysis Consortium lung adenocarcinoma (CPTAC-LUAD) dataset and collected the information on 178 CT, 178 PET, and the patients' age, history of smoking, and gender. We conducted image processing and feature extraction. Finally, 4 computed tomography (CT) image features and 2 positron emission tomography (PET) image features were extracted. Four prediction models based on CT image features, PET image features, and demographic data were developed, and the area under the receiver operating characteristic (ROC) curve was used to evaluate the performance of prediction models. A total of 178 eligible samples were randomly divided into a training set (*n* = 134) and a testing set (*n* = 44) at a ratio of 3 : 1, with 2021 as a random number. ROC analyses illustrated that the predictive performance for distant metastases of combining CT-PET image features and demographic data for training and testing were 0.923 (95% confidence interval (CI): 0.873–0.973) and 0.873 (95% CI: 0.757–0.990). In addition, the predictive performance of the combined model in the testing set was significantly better than that of the CT-demographic data model (0.716, 95% CI: 0.531–0.902), PET-demographic data model (0.802, 95% CI: 0.633–0.970), and CT-PET model (0.797, 95% CI: 0.666–0.928). The random forest model via combining CT-PET image features and demographic data could have great performance in predicting distant metastases among lung cancer patients.

## 1. Introduction

Lung cancer, one of the most common cancers, has been recognized as the leading cause of cancer-related deaths all over the world, and an estimated 2.20 million new cases and 1.76 million deaths occur each year [1]. Several studies showed that metastasis was a major cause of deaths in most patients with cancer [2, 3]. The distant metastases of advanced lung cancer are very extensive and could spread in various parts of the body, such as the lungs, liver, brain, and bone, which pose a serious threat to the life and health of patients [4]. Not only that, the treatment method for lung cancer patients is also associated with the staging of distant metastases [5]. Therefore, accurate determination of the distant stage for lung cancer plays an important role in evaluating tumor prognosis for patients.

Nowadays, the diagnosis of distant metastasis in lung cancer is mainly based on digital imaging, such as computed tomography (CT) [4], magnetic resonance imaging (MRI) [6], positron emission tomography (PET) [7], and ^18^F-fluorodeoxyglucose PET/CT (^18^FDG PET/CT) [8]. Aided diagnosis has important research significance and application value in accurately screening lesions, reducing the rate of missed diagnosis and misdiagnosis, reducing labor intensity, and improving the efficiency of reading films [9]. At present, several studies have developed risk prediction models to estimate the risk of lung cancer patients [10, 11] and pointed out sociodemographic information related to risk of lung cancer, including age, history of smoking, and gender.

As far as we know, most studies have only used these digital imaging technologies to diagnose distant metastases in lung cancer patients until now [7, 12], and there are few studies that combine demographic information to make a diagnosis. In addition, in the early diagnosis and curative effect evaluation of lung cancer, PET/CT fusion image with high sensitivity and specificity could not only realize the function of molecular imaging and anatomical imaging fusion, which is a kind of “positive” whole-body imaging method, but also reflect the pathological changes and organization structure and pathological changes of the lesion area [13, 14]. Herein, this study mainly aimed to develop and validate a random forest model of PET/CT image features combined with demographic data to diagnose distant metastases among lung cancer patients.

## 2. Materials and Methods

### 2.1. Data Sources and Collection

The patients' information in this study was obtained from The Cancer Genome Atlas Lung Adenocarcinoma (TCGA-LUAD) dataset (*n* = 12), the lung PET-CT dataset (*n* = 132), the lung squamous cell carcinoma (LSCC) dataset (*n* = 6), and the National Cancer Institute's Clinical Proteomic Tumor Analysis Consortium lung adenocarcinoma (CPTAC-LUAD) dataset (*n* = 2). A total of 178 CT and 178 PET were collected in this study. Simultaneously, the patients' demographic information, such as age, history of smoking, and gender, was also extracted from these datasets.

### 2.2. Feature Extraction

**Figure 1:**
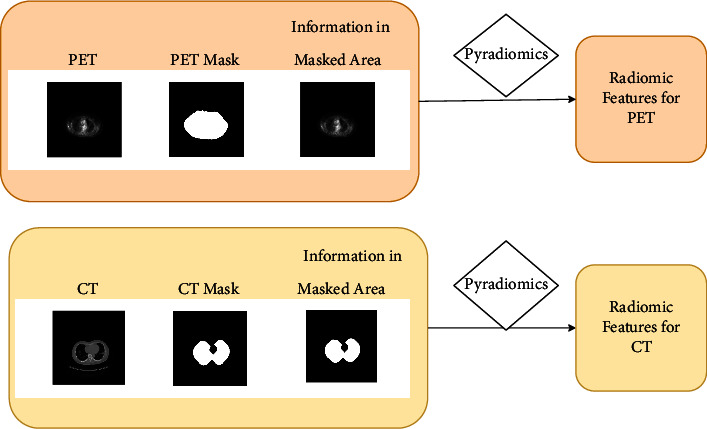
Image processing of CT and PET.

In this study, all images were resized to 512 × 512 (Figure 1), and then, lung masks were extracted and made according to HU values of −900∼−700. Ndimage was used to fill possible gaps in the lungs, and skimage morphology was used to expand and ensure coverage of all lung areas. CT images, PET images, and their corresponding masks obtained from preprocessing were extracted by pyradiomics, including 18 first-order statistics, 10 two-dimensional shape features, and 75 texture features including (the gray-level co-occurrence matrix (GLCM, 24), the gray-level run length matrix (GLRLM, 16), the gray-level size zone (GLSZM, 16), the neighborhood gray-tone difference matrix (NGTDM, 5), and the gray-level difference method (GLDM, 14)). The features of CT and PET images were screened by Lasso regression, and finally, 4 CT image features and 2 PET image features were screened out.

### 2.3. Statistical Analysis

Measurement data were tested for normality by the Shapiro Test, the continuous variables of normal distribution were expressed by the mean ± SD, and the comparison between groups was tested by the *T* test. The measurement data of non-normal distribution were expressed by the median and interquartile distance, and the Mann–Whitney *U* test was used to compare between groups. Counting data were described by the number of cases and the composition ratio *N* (%), and the comparison between groups adopted the *χ*^2^ test or Fisher's exact probability method. Missing data were interpolated multiple times (Supplementary Table 1), SAS 9.4 statistical analysis software and Python analysis were used, and all statistical tests were conducted by two-sided tests. The difference between the tests was statistically significant at *P* < 0.05.

**Figure 2:**
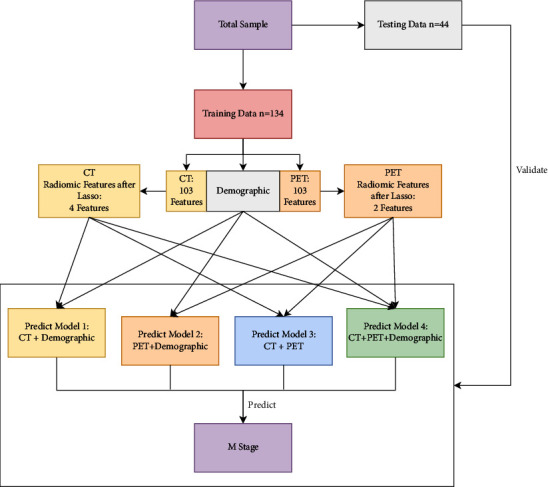
Flowchart of the analysis method.

In the present study, 178 samples were randomly divided into a training set for developing prediction models and a test set for validating the models at a ratio of 3 : 1, with 2021 as a random number. Then, sensitivity analysis of demographic data was carried out. We constructed four prediction models based on CT image features, PET image features, and demographic data: (1) the random forest model of CT image features combined with demographic data, (2) the random forest model of PET image features combined with demographic data, (3) the random forest model of CT combined with PET, and (4) the random forest model of CT-PET image characteristics combined with demographic data. The flowchart of the analysis method is shown in Figure 2. The area under the receiver operating characteristic (ROC) curve was used to evaluate the performance of the prediction models.

## 3. Results

### 3.1. Patient Characteristics

A total of 178 eligible samples were enrolled, with 134 samples in the training set and 44 samples in the testing set. The average age was 64.56 and 62.41 years in the training and test datasets, respectively. There was no significant difference in demographic data between the training set and the testing set, which showed that our data balance is comparable (Supplementary Table 2).

After screening by Lasso regression, we screened out 4 CT and 2 PET image features, of which 4 CT image features included first-order 10 percent, first-order robust mean absolute deviation, GLCM joint average, and NGTDM strength; 2 PET image features contained GLDM low gray-level emphasis and NGTDM strength. Subsequently, we compared demographic data, 4 CT image features, and 2 PET image features in the training set (Table 1). In the comparison between groups, we found that the number of smokers in the M0 stage was 78 (71.56%) more than that in the M1 stage (10 (40.00%)). Among the image features, the value of low gray-level emphasis extracted from PET in M1 (0.30 (0.23, 0.33)) was greater than the value in M0 (0.18 (0.07, 0.32)).

### 3.2. Development and Visualization of the Prediction Model

Demographic data (age, history of smoking, and gender) were included in the three prediction models. In the CT-related random forest model, we included CT image features (first-order 10 percent, first-order robust mean absolute deviation, GLCM joint average, and NGTDM strength) and demographic data; GLDM low gray-level emphasis, NGTDM strength, and demographic data were included in the PET-related random forest model. CT-PET image features were included in the CT-PET random forest model. Additionally, we also established a random forest model by combining CT-PET image features with demographic data.

**Figure 3:**
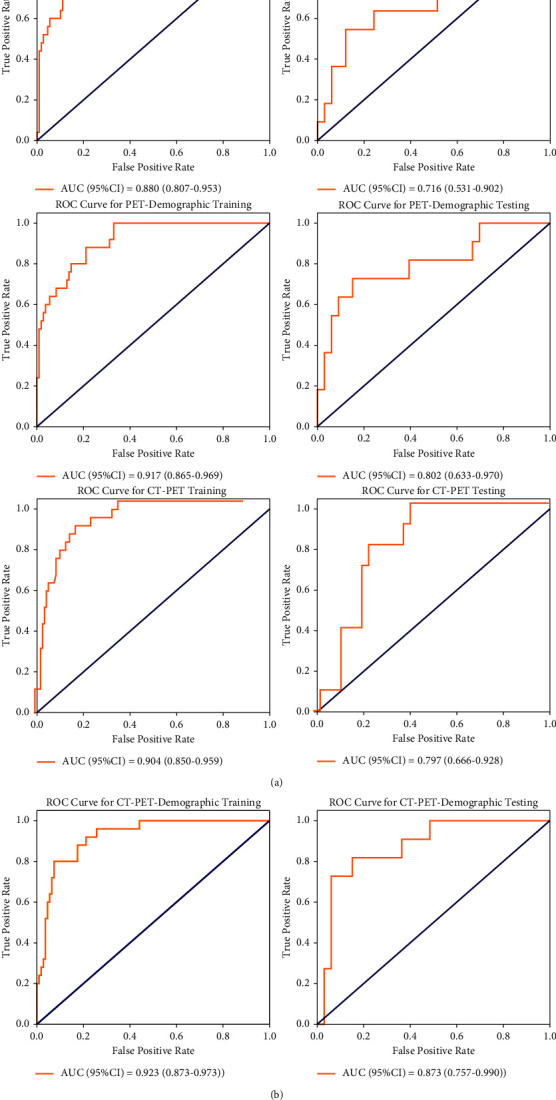
The ROC curves of the four random forest models.

**Figure 4:**
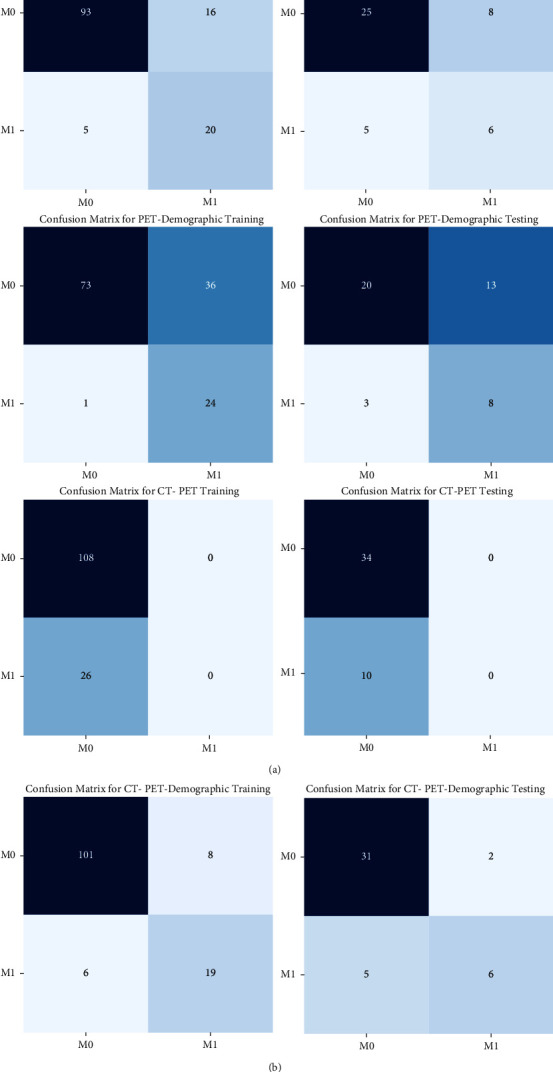
The confusion matrix of the four random forest models in the training and testing sets.

**Figure 5:**
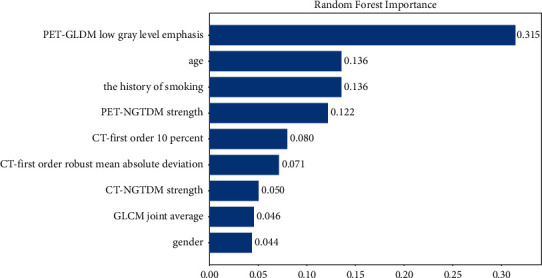
Random forest importance of the CT-PET-demographic data model.

The performance of random forest models in the training set and testing set is shown in Table 2. The area under the curve (AUC) of the CT-PET demographic data model was 0.923 (95% CI: 0.873–0.973) in the training set, which was higher than that of the CT demographic data model (0.880, 95% CI: 0.807–0.953), PET demographic data model (0.917, 95% CI: 0.865–0.969), and CT-PET model (0.904, 95% CI: 0.850–0.959). Similar results were observed in the testing set. These results also indicated that CT-PET combining demographic data was the best model to predict the M stage among the four prediction models established by using the random forest. The ROC curves and the confusion matrix of four models are shown in Figures 3 and 4, respectively. Furthermore, Figure 5 also depicts the variable importance of features for the random forest model of CT PET-demographic data. GLDM low gray-level emphasis, which was extracted from PET, was the most important of the nine factors, followed by age and the history of smoking in demography.

## 4. Discussion

Lung cancer is still one of the leading causes of cancer death worldwide, with distant metastasis accounting for the majority of deaths [15]. Not only that, early detection of distant metastases in lung cancer patients could provide an effective basis for the formulation of clinical treatment, which is also the focus of current clinical research [16]. In the present study, we aimed at developing a random forest model to diagnose distant metastases in lung cancer patients by combining the characteristics of CT-PET images with demographic data. The performance of this prediction model was internally validated at the same time. Our results indicated that the random forest model created by combining CT-PET images and demographic data was effective in predicting distant metastases in lung cancer patients. Age, history of smoking, gender, first-order 10 percent, first-order robust mean absolute deviation, GLCM joint average, NGTDM strength (CT), GLDM low gray-level emphasis, and NGTDM strength (PET) were important factors for predicting distant metastases in lung cancer patients.

In recent years, with the rapid development of imaging technology, CT, PET, and PET/CT examinations have been gradually applied to the diagnosis of malignant tumors, which can provide more information related to anatomical structure and tissue metabolism, and have higher value in the qualitative diagnosis of lesions [17–19]. Despite the fact that CT examination could intuitively reflect the morphological characteristics of lesions and carry out accurate anatomical positioning, it has great limitations in diagnosing distant metastases in patients based on morphology [20]. Several studies have pointed out that PET/CT, as a tool that can effectively integrate anatomical, morphological, and biological information of lesions, has gradually become widely used in clinical practice [21, 22]. In the study by Yu et al., they confirmed that ^18^F-FDG PET/CT has a good diagnostic performance for distant metastasis staging in patients with non-small-cell lung cancer (NSCLC) at initial staging [23]. More importantly, some demographic data were also considered, as this could influence the morbidity and distant metastases of lung cancer.

In this study, we extracted the characteristics of CT images (first-order 10 percent, first-order robust mean absolute deviation, GLCM joint average, and NGTDM strength) and PET images (GLDM low gray-level emphasis and NGTDM strength) and collected the patients' demographic data (age, history of smoking, and gender). Thus, we constructed four prediction models: combining CT image features and demographic data; combining PET image features and demographic data; combining CT and PET image features; and combining CT image features, PET image features, and demographic data. Through ROC curve analysis, we found that the model combining CT and PET image features and demographic data may have a better predictive performance in predicting distant metastases in lung cancer patients than those models combining CT image features and demographic data, combining PET image features and demographic data, and combining CT and PET image features. Previous studies have revealed that age, history of smoking, and gender are associated with the distant metastasis risk of lung cancer patients [24, 25]. In a study examining the effects of smoking on brain metastases in lung cancer patients, Wu et al. showed that the incidence of brain metastases was significantly higher in smokers than in those who had never smoked [26]. The potential reason was that nicotine, as the main cigarette ingredient, might promote brain metastases by distorting the polarity of M2 microglia, which in turn accelerates the growth of metastatic tumors. Our results were consistent with those of previous research studies, in which the variable importance of features in the random forest model showed that age, gender, and history of smoking in demography were the important risk factors. To the best of our knowledge, this is the first study to assess the value of CT-PET imaging combined with demographic data for diagnosis of distant metastases in lung cancer patients. We believed that the finding could provide clinicians with more convenience in diagnosing distant metastases of lung cancer and making personalized treatment strategies.

The study has several limitations. First, the study had a relatively small sample size, which might have limited statistical power. Second, owing to all patients' information being derived from the TCGA-LUAD, lung PET-CT, LSCC, and CPTAC-LUAD datasets, we did not collect data on the race and types of lung cancer, which might be associated with the distant metastases of lung cancer [27]. More research studies are needed to explore this association. Third, there was no external validation, and larger datasets are needed to further confirm our findings.

## 5. Conclusion

In conclusion, our study displayed that the random forest model created by combining CT-PET image features and demographic data could have great performance in predicting distant metastases in lung cancer patients. The developed model may provide supplementary guidance for clinicians in the choice of therapeutic strategies and personalized monitoring for lung cancer patients.

## Figures and Tables

**Table 1:** Comparison of demographic data, 4 CT image features, and 2 PET image features among the M0 and M1 groups.

Variables	Total (*n* = 134)	M0 (*n* = 109)	M1 (*n* = 25)	Statistic	*P*
History of smoking, *n* (%)				*χ* ^2^ = 8.985	0.003
No	46 (34.33)	31 (28.44)	15 (60.00)		
Yes	88 (65.67)	78 (71.56)	10 (40.00)		
Gender, *n* (%)				*χ* ^2^ = 0.486	0.486
Male	72 (53.73)	57 (52.29)	15 (60.00)		
Female	62 (46.27)	52 (47.71)	10 (40.00)		
Age, mean ± SD	64.56 ± 9.39	64.80 ± 9.01	63.49 ± 11.03	*t* = 0.63	0.529
CT					
First-order 10 percent, M (*Q*_1_, *Q*_3_)	136.60 (117.00, 165.00)	134.00 (117.00, 161.00)	140.00 (119.00, 199.00)	*Z* = 0.768	0.442
First-order robust mean absolute deviation, mean ± SD	357.41 ± 25.63	358.68 ± 24.12	351.87 ± 31.35	*t* = 1.20	0.233
GLCM joint average, mean ± SD	23.99 ± 2.77	23.86 ± 2.69	24.55 ± 3.10	*t* = −1.13	0.262
NGTDM strength, M (*Q*_1_, *Q*_3_)	3.12 (2.63, 3.84)	3.12 (2.60, 3.84)	3.40 (2.76, 3.74)	*Z* = 0.942	0.346
PET					
GLDM low gray-level emphasis, M (*Q*_1_, *Q*_3_)	0.22 (0.08, 0.32)	0.18 (0.07, 0.32)	0.30 (0.23, 0.33)	*Z* = 2.547	0.011
NGTDM strength, M (*Q*_1_, *Q*_3_)	2909.75 (174.97, 12124.24)	2714.84 (179.85, 9674.76)	6176.46 (128.30, 14465.71)	*Z* = 0.463	0.644

*Note.* CT: computed tomography; PET: positron emission tomography; GLCM: gray-level co-occurrence matrix; NGTDM: neighborhood gray-tone difference matrix; GLDM: gray-level difference method.

**Table 2:** The performance of the three random forest models.

Random forest models	Dataset	PPV (95% CI)	NPV (95% CI)	AUC (95% CI)	Accuracy (95% CI)
CT demographic data	Training set	0.556 (0.393–0.718)	0.949 (0.905–0.993)	0.880 (0.807–0.953)	0.843 (0.782–0.905)
CT demographic data	Testing set	0.429 (0.169–0.688)	0.833 (0.700–0.967)	0.716 (0.531–0.902)	0.705 (0.570–0.839)
PET demographic data	Training set	0.410 (0.286–0.533)	1.000 (1.000–1.000)	0.917 (0.865–0.969)	0.731 (0.656–0.806)
PET demographic data	Testing set	0.381 (0.173–0.589)	0.870 (0.732–1.000)	0.802 (0.633–0.970)	0.636 (0.494–0.779)
CT-PET	Training set	0.523 (0.375–0.670)	0.967 (0.930–1.000)	0.904 (0.850–0.959)	0.821 (0.756–0.886)
CT-PET	Testing set	0.364 (0.079–0.648)	0.818 (0.687–0.950)	0.797 (0.666–0.928)	0.705 (0.570–0.839)
CT- PET demographic data	Training set	0.714 (0.547–0.882)	0.953 (0.912–0.993)	0.923 (0.873–0.973)	0.903 (0.853–0.953)
CT-PET demographic data	Testing set	0.750 (0.450–1.000)	0.861 (0.748–0.974)	0.873 (0.757–0.990)	0.841 (0.733–0.949)

*Note.* CT: computed tomography; PET: positron emission tomography; CI: confidence interval; PPV: positive predictive value; NPV: negative predictive value; AUC: area under the curve.

## Data Availability

The data utilized to support the findings are available from the corresponding authors upon request.

## Supplementary Materials

Supplementary Materials Supplementary Table 1: missing value filling and sensitivity analysis. Supplementary Table 2: balance test.
