# Supplementary material for: Development and Validation of the Random Forest Model via Combining CT-PET Image Features and Demographic Data for Distant Metastases among Lung Cancer Patients
Source: J Healthc Eng. 2022 Dec 13;2022:7793533. doi: 10.1155/2022/7793533 (PMC9767733; doi:10.1155/2022/7793533)
Supplement: Supplementary Materials — Supplementary Table 1: missing value filling and sensitivity analysis. Supplementary Table 2: balance test. [file 7793533.f1.docx]

**Supplemental Table 1 Missing value filling and sensitivity analysis**

| Missing variable | Missing proportion | Before filling | After filling | Statistic | *P* |
| --- | --- | --- | --- | --- | --- |
| History of smoking | 16 (8.99%) |  |  | χ^2^=0.049 | 0.824 |
| No |  | 62 (34.83%) | 65 (36.52%) |  |  |
| Yes |  | 100 (61.72%) | 113 (63.48%) |  |  |

**Supplemental Table 2 Balance test**

| Variables | Total (n=178) | Training set (n=134) | Testing set (n=44) | Statistic | *P* |
| --- | --- | --- | --- | --- | --- |
| History of smoking, n (%) |  |  |  | χ^2^=1.120 | 0.290 |
| No | 65 (36.52) | 46 (34.33) | 19 (43.18) |  |  |
| Yes | 113 (63.48) | 88 (65.67) | 25 (56.82) |  |  |
| Gender, n (%) |  |  |  | χ^2^=0.127 | 0.721 |
| Female | 81 (45.51) | 62 (46.27) | 19 (43.18) |  |  |
| Male | 97 (54.49) | 72 (53.73) | 25 (56.82) |  |  |
| Age, Mean ± SD | 64.03 ± 9.37 | 64.56 ± 9.39 | 62.41 ± 9.25 | t=1.32 | 0.189 |
| M Stage, n (%) |  |  |  |  |  |
| M0 | 142 (79.78%) | 109 (81.34%) | 33 (75.00%) | χ^2^=0.480 | 0.489 |
| M1 | 36 (20.22%) | 25 (18.66%) | 11 (25.00%) |  |  |
